# Human Bocavirus – Insights into a Newly Identified Respiratory Virus

**DOI:** 10.3390/v1010003

**Published:** 2009-04-21

**Authors:** Jessica Lüsebrink, Felix Wittleben, Verena Schildgen, Oliver Schildgen

**Affiliations:** Institute of Virology, University of Bonn, Sigmund-Freud-Straße 25, D-53105 Bonn, Germany E-Mails: JessicaLuesebrink@googlemail.com (J.L.), Felix.Wittleben@googlemail.com (F.W.), Verena.Schildgen@freenet.de (V.S.)

**Keywords:** Bocavirus, Respiratory virus, HBoV

## Abstract

Human Bocavirus (HBoV) was discovered in 2005 using a molecular virus screening technique. It is often found in respiratory samples and is a likely cause for respiratory diseases in children. HBoV is distributed worldwide and has been found not only in respiratory samples, but also in feces, urine and serum. HBoV infections are mostly found in young children and coinfections with other respiratory viruses are often found, exacerbating the efforts to link HBoV to specific symptoms. The purpose of this review is to give an overview of recent HBoV research, highlighting some recent findings.

## 1. Discovery

Human bocavirus (HBoV) was discovered in 2005 by Allander *et al*. [1] in respiratory samples from children with suspected acute respiratory tract infection (ARTI) using a novel technique. This molecular virus screening is based on a random PCR-cloning-sequencing approach and was employed on two chronologically distinct pools of nasopharyngal aspirates (NPAs). It revealed a parvovirus-like sequence, with close relation to the members of the bocavirus genus.

A retrospective study revealed 17 (3.1 %) out of 540 NPAs positive for HBoV, with 14 specimens tested negative for other viruses, giving the suggestion that HBoV is a causative agent of respiratory tract infections [1].

## 2. Taxonomy

HBoV is a putative member of the family *Parvoviridae*, subfamily *Parvovirinae*, genus *Bocavirus*. Before identification of HBoV, parvovirus B19 of the genus *Erythrovirus* was the only known human pathogen in the family of parvoviruses. Parvovirus B19 is widespread and manifestations of infection vary with the immunologic and hematologic status of the host. In immunocompetent children, parvovirus B19 is the cause for erythema infectiosum. In adults it has been associated with spontaneous abortion, non-immune hydrops fetalis, acute symmetric polyarthropathy, as well as several auto-immune diseases [2,3,4,5].

Based on its genomic structure and amino acid sequence similarity shared with the namesake members of the genus, bovine parvovirus (BPV) and canine minute virus (MVC), HBoV was classified as a bocavirus and therefore provisionally named human bocavirus [1].

Other subfamily *Parvovirinae* members known to infect humans are the apathogenic adeno-associated viruses of the genus *Dependovirus* and parvovirus 4 [6,7]. Parvovirus 4 has not yet been assigned to a genus, but it was proposed to allocate it to the genus *Hokovirus* as it shares more similarities to the novel porcine and bovine hokoviruses than with other parvoviruses [8]. Recently a second human bocavirus has been identified, HBoV2, with 75.6 % nucleotide similarity to HBoV [9]. HBoV2 was found in stool samples from Pakistani children as well as in samples from Edinburgh (1 of the 3 positive samples was derived from a patient >65 years old), indicating that it is not restricted to one region or to young children.

## 3. Virion structure and genome organization

The parvoviridae are small, non-enveloped viruses. The isometric nucleocapsids with diameters of 18 to 26 nm contain a single molecule of linear, negative-sense or positive-sense, single stranded DNA with an average genome size of 5,000 nucleotides.

A study on the polarity of the packaged strand confirms that HBoV replication leads to packaging of single stranded DNA in the majority of cases. By using the NASBA methods, Böhmer *et al*. showed that negative strands were packaged in 87.5 % of the investigated samples [10].

The complete genome of HBoV has yet to be determined. Until today at least 5,309 nt were identified (GeneBank Accession-Number EU 984244). The genome of other parvoviruses is flanked by palindromic hairpin structures essential for DNA replication and it can be assumed that this is also true for HBoV. The hairpin structures of HBoV could not be deciphered by sequencing methods so far and the complete sequence of the genome remains unknown until the flanking structures are elucidated.

Three open reading frames (ORF) can be found in the genome of HBoV, similar to BPV and MVC. One ORF encodes a non-structural protein (NS1) and a second one at least two capsid proteins (VP1 and VP2). The third ORF encodes a non-structural protein (NP1). The function of HBoV NS1 is unknown. In MVC and minute virus of mice, NS1 is a multifunctional protein, essential for viral DNA replication [11,12]. Furthermore, a role in apoptosis, cell cycle arrest, and transactivation of cellular genes has been described for parvovirus B19 NS1 [13,14,15,16]. NP1 is absent in other parvoviruses and, like for NS1, the function of HBoV NP1 is unknown. In MVC, NP1 plays an essential role in DNA replication [11]. Cross-complementation tests with NP1 of MVC, BPV, and HBoV showed that they all could increase DNA replication in NP1 knockout mutants, suggesting they all have analog functions [11].

Alignment studies showed that amino acid variations seem to appear mostly in the genes of the capsid proteins while NS1 and NP1 represent the most conserved regions of the HBoV genome [17], reflecting the more immunogenic character of the virion-associated proteins.

## 4. Laboratory diagnosis

HBoV detection has been mostly performed on NPAs and swabs and relies mostly on classical [1,18,19,20,21,22,23] and real-time PCR [24,22,25,26,27,28]. Real-time PCR sure has advantage over the conventional PCR, as it offers greater sensitivity, specifity, and reduced expenditure of time. PCR assays detecting the NS1 or NP1 gene are most common. Tozer *et al*. established a highly sensitive real-time PCR assay targeting the NP1 and the VP1 gene and were able to detect HBoV in respiratory samples, as well as in fecal samples and whole blood [29].

Additional to the PCR assays, HBoV can be detected indirectly via detection of antibodies to HBoV. This method has also been performed with different ELISAs using virus-like-particles (VLP) of HBoV VP1 or VP2 [30,31,32,33]. VLPs were produced by using an insect cell line infected with a baculovirus expression vector. These VLPs were then used to produce rabbit anti-serum with high titers of immunoglobulins specific for HBoV, which could be used in the ELISA. All established ELISAs were able to detect anti-HBoV antibodies in sera.

## 5. Epidemiology

After the first description of HBoV, it has been reported from various countries, pointing to a worldwide endemicity. HBoV has been identified in Europe [34,35,36,37,38,39,40], Asia [41,42,24,43], Australia [18,44,23], Africa [25] and America [45,19,46,20]. Until now, it is known that HBoV exists as a single lineage with two different genotypes [47]. The prevalence of HBoV ranges between 1.5 to 19.3 % [19,48]. Primary infection with HBoV seems to occur early in life and children between the ages of six and 24 months seem to be mostly affected [31,49,43,22,50], but older children can be infected, too. Newborns may be protected by antibodies against HBoV derived from the mothers [43]. HBoV-IgG is able to cross the placenta to the fetus, but it remains unclear if vertical maternal-fetal transmission occurs [51]. HBoV infections are rarely found in adults [52,21,53,54]. Lindner et al. detected anti-HBoV antibodies in 94 % of healthy blood donors > 19 years of age. Seronegative individuals were mostly found in children between the ages of 1 and 3 years, while the seroprevalence starts to increase in children aged 5 to 10 years, further indicating that the first infection with HBoV happens in the first years of life.

A seasonal distribution of the virus has not yet been clearly demonstrated. HBoV has been detected throughout the whole year, with peak seasons varying from year to year and from study to study. However, HBoV has been detected mostly in fall and winter months [39,55,56,57].

## 6. HBoV and respiratory tract diseases

As HBoV has been first identified in respiratory samples, it has been suggested as a respiratory tract infection agent [1]. The majority of the following studies in fact detected HBoV in children with respiratory tract infections. Clinical symptoms mostly described in conjunction with an HBoV infection are wheezing, fever, bronchiolitis and pneumonia [28,49,36,50]. Studies including asymptomatic controls showed that HBoV is also detectable in these controls but with a lower incidence [20,58,59]. For example, HBoV was detected in 17 % of children hospitalized because of respiratory infection, while only 5 % of the surveyed asymptomatic children were HBoV positive [58]. This supports the assumption that HBoV in fact could be assigned to the respiratory viruses.

In contrast to other studies, in the study of Longtin *et al*. 43 % of asymptomatic children tested positive for HBoV [53]. Most of those children underwent myringotomies, adenoidectomies or tonsillectomies. Thus, Lu *et al*. suggested that HBoV may be present in tonsillar lymphocytes [60]. They tested DNA extracts of lymphocytes from nasopharyngeal tonsils or adenoids and palatine/lingual tonsils. 32.3 % of the tested extracts were HBoV positive, indicating that HBoV establishes latent or persistent infection.

Coinfections with other viruses are frequently observed in HBoV infections and often occur in more than 50 % of the tested samples [56,46,61]. Two recent studies report that the viral load of HBoV was significantly higher in children with monoinfections than in children with coinfections [57,62]. The high rate of coinfections with other viruses may then be explained by the persistence of HBoV in the respiratory tract. DNA quantification in HBoV positive samples revealed that the viral load of 42.5 % of the positive patients was > 1.0 × 10^5^ DNA copies/mL, suggesting that below this cut-off HBoV may be a persistant virus or a bystander [54].

## 7. HBoV and gastroenteric infections

MVC and BPV, the two other members of the genus bocavirus are also known to cause gastrointestinal infections in dogs and calves, respectively [63,64]. Several studies detected HBoV in stool samples from children with acute gastrointestinal illness [65,66,67,38], but the role of HBoV infection in the gastrointestinal tract is still unclear. A study on the role of HBoV in gastroenteritis outbreaks in day care facilities detected HBoV in 4.6 % of 307 stool samples. Coinfections with Norovirus were frequent [68]. Another study on hospitalized children with acute gastroenteritis also reports a high coinfection rate with other gastroenteritic viruses [69]. Both reports could not link HBoV to gastroenteritis in children, indicating that HBoV may not be a causative agent. The gastrointestinal epithelium may instead be the place of HBoV replication.

Besides HBoV detection in respiratory samples and feces, HBoV DNA was also found in serum/whole blood [28,59,29] and one study reports detection in urine [70]. As there are no currently established methods to detect HBoV particles, it remains unclear if the detection of HBoV in serum indicates viremia or if HBoV targets blood cells. Parvovirus B19 infects erythroid progenitor cells in the bone marrow [2], but HBoV DNA was not detected in bone marrow of HIV (human immunodeficiency virus)-infected and HIV-uninfected individuals, while parvovirus B19 was detected in both groups [71].

## 8. Immunology

As most of the published data regarding HBoV are prevalence studies, the immunologic response to HBoV remains unclear. The work of Chung *et al*. gives a first insight into the cytokine response to HBoV. They observed that interferon-γ, interleukin-2 and interleukin-4 are elevated in HBoV positive specimens compared to the controls [72]. In comparison to respiratory syncytial virus infected children, HBoV infected children showed lower levels of tumor necrosis factor-α and interleukin-10.

## 9. Outlook

It remains unclear how far HBoV contributes to respiratory and/or gastrointestinal disease. More and more evidence supports the assumption that HBoV is indeed an infectious and contagious agent, but a chance remains that it solely synergistically increases the clinical severity of other infections. Consequently, well planned and designed clinical studies with sophisticated case controls need to be performed in order to finally rule out the role of bocavirus, unless animal or at least in vitro models demonstrate its pathogenicity.
